# Habitat Utilization Preference by Small Mammals Is Associated With Geomorphic and Soil Properties: A Case Study of the Plateau Pika From the Eastern Qinghai‐Tibet Plateau

**DOI:** 10.1002/ece3.73812

**Published:** 2026-06-08

**Authors:** Faming Qin, Yingyuan Chen, Zhongmin Tang, Maria K. Oosthuizen, Shenghui An, Wanrong Wei

**Affiliations:** ^1^ Key Laboratory of Southwest China Wildlife Resources Conservation, College of Life Sciences China West Normal University Nanchong China; ^2^ Liziping Giant Panda's Ecology and Conservation Observation and Research Station of Sichuan Province Nanchong China; ^3^ Gannan Grassland Workstation in Gansu Province Hezuo China; ^4^ Mammal Research Institute University of Pretoria Hatfield South Africa; ^5^ Forestry, Animal Husbandry and Grassland Science Gannan Tibetan Academy of Agriculture Hezuo China; ^6^ State Key Laboratory of Grassland Agro‐Ecosystems, College of Pastoral Agriculture Science and Technology Lanzhou University Lanzhou China

**Keywords:** generalized additive model, geomorphic characteristics, habitat utilization preferences, plateau pika, principal component analysis, soil characteristics

## Abstract

Amid accelerating climate change, understanding how wildlife adjusts to environmental constraints, particularly via habitat use, is essential. In high‐altitude regions with extreme environmental conditions and strong ecological filtering effects, geomorphic and soil characteristics are critical factors influencing habitat utilization preferences. Previous studies of factors affecting wildlife habitat‐use preferences have primarily focused on low‐altitude areas. However, research remains scarce on the relationship between habitat utilization preferences of small mammals in plateau regions and geomorphic and soil characteristics. In this study, we selected 66 plots from 19 typical distribution areas of pikas in three counties of the Qinghai‐Tibetan Plateau to assess the relationship between the pika's habitat‐utilization preferences and geomorphic and soil characteristics at a large scale. The results revealed that the factors affecting the habitat use of pikas are potentially similar across regions. The resource selection index (*Ei*) showed a clear preference for specific geomorphic and soil characteristics. In addition, we found a significant correlation between PC1–PC4 and pika density, and both principal component analysis (PCA) and generalized additive models (GAM) indicated that all geomorphic and soil properties, except soil depth, affect the habitat use of pikas. Overall, pikas preferred flat terrain with a wide field of view (“beach”) and sunny, south‐facing slopes; including running water sources, with the distance between the preferred habitats to water resources related to the type of water resources. In addition, pikas preferred loam (*Ei* = 0.258), soil depths > 40 cm (*Ei* = 0.214) but < 60 cm (*Ei* = 0.241), soil organic matter between 5% and 15% (*Ei* = 0.359) and soil moisture between 10% and 20% (*Ei* = 0.290). Our results highlight that the habitat‐utilization preferences of pikas are highly dependent on geomorphic and soil characteristics. Future analyses of factors affecting habitat utilization preferences for small mammals should take geomorphic and soil characteristics into consideration.

## Graphical abstract

This study, through a large‐scale survey, reveals a close relationship between plateau pika habitat use preferences and geomorphic and soil characteristics. The findings indicate that pikas prefer flat, sun‐facing terrain near water sources, as well as specific ranges of soil depth, organic matter, and moisture. These preferences represent behavioral adaptation strategies employed in response to environmental changes.

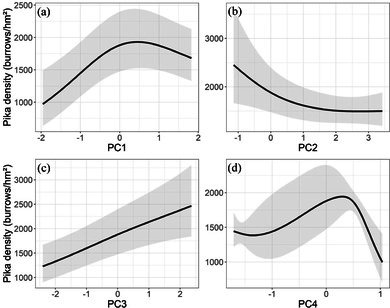

## 1 Introduction

Against the backdrop of accelerating global climate change, wildlife faces unprecedented environmental challenges and survival pressures (Parmesan 2006; Fatima and Irshad 2025). In response, mammals employ behavioral and physiological adjustments to ensure survival and reproduction (Tuomainen and Candolin 2011). Habitat utilization preferences are an important manifestation of animal behavioral and physiological adaptation, reflecting how animals optimize their living space through mobility and decision‐making to cope with changing environments (Huey 1991; Tuomainen and Candolin 2011; Sih et al. 2011).

Numerous factors influence the habitat use of animals, including physiological and morphological characteristics of the animals, food availability, predation risk, and interspecific competition, and a variety of habitat characteristics such as geomorphology, proximity to water, and soil characteristics (Doligez et al. 2002; Mayor et al. 2009). The habitat use of wild animals is a complex multi‐dimensional system in which multiple ecological factors determine the degree of habitat utilization, and the relationships among these ecological factors are equally complex (Bjørneraas et al. 2012). Broadly, two categories of factors affect habitat utilization preferences. Predation affects survival and fitness and can therefore shape habitat use (Madhusudhan and Johnsingh 1998). In addition, environmental factors play a critical role in determining habitat quality. Environmental characteristics such as landform, terrain, access to water, and soil composition are important considerations that cannot be ignored in understanding how animals utilize their habitats (Madhusudhan and Johnsingh 1998).

Understanding the factors that influence wildlife habitat utilization preferences is important for interpreting how animals adapt to changing environments (Tuomainen and Candolin 2011). Among the various influencing factors, geomorphic and soil characteristics are the primary external environmental drivers of wildlife habitat utilization preferences and serve as important indicators of habitat use intensity (Lunghi et al. 2015; Aneta and Krzysztof 2020). Many studies report on the geomorphology and soil characteristics of wild animal utilization that are distributed in forests and low‐elevation grasslands (Araújo et al. 2008), such as Felidae (Wei et al. 2000; Meloro et al. 2013), Cercopithecidae (McGraw 1998; Wu et al. 2004; Wang et al. 2018), and Sciuridae (Avila‐Flores et al. 2010; Olimb et al. 2022), and these studies found that the terrain (slope and orientation) and water sources are key factors affecting the use of animal habitats. Unlike low altitude areas, harsh habitat conditions in high‐altitude areas may have a stronger filtering effect on the utilization of small mammals' habitats (Kamenišťák et al. 2020). However, studies focusing on the relationship between the geomorphic and soil characteristics and habitat utilization preferences for small mammals in high plateau areas are relatively scarce.

**FIGURE 1:**
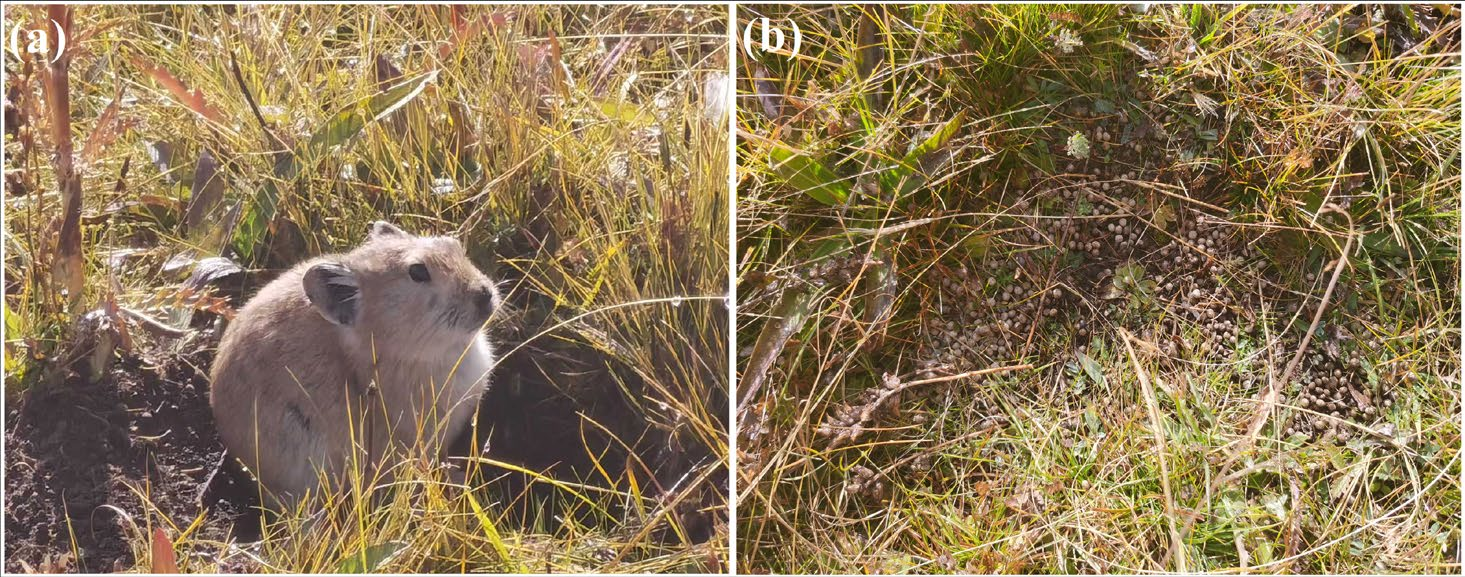
The plateau pika ( *Ochotona curzoniae* ) (a) and its feces in alpine meadows (b).

The plateau pika ( *Ochotona curzoniae* ), also known as the black‐lipped pika, is a small herbivorous mammal common in the Qinghai‐Tibet Plateau (QTP) (Wei et al. 2019). The plateau pika (hereinafter referred to as pika) is a keystone species and ecosystem heterologous engineer (Figure 1) because pikas are an important food source for carnivores and can increase plant species diversity, plant productivity, water infiltration (Wilson and Smith 2015), soil organic carbon and nitrogen and nutrient cycling rates (Lai and Smith 2003; Pang and Guo 2017; Wei et al. 2023), and play an important positive role in the maintenance of the balance and stability of the grassland ecosystem when their density is low (Wei, Zhen, et al. 2020; Wei et al. 2023). However, the pika is also regarded as one of the most important biological pest species because it is perceived to compete for food with livestock and create bare soil patches that increase soil erosion when pika population densities exceed a certain threshold (Wei et al. 2023; Wang, Yan, Martin, et al. 2024; Qin et al. 2026). At present, studies on the pika mainly focus on its habits, physiology, and influences on grassland vegetation and soil properties (Zhang, Liu, and Wang 2005; Aurélien et al. 2013; Pang and Guo 2017; Zhou et al. 2023; Wang, Qian, Zhu, et al. 2024; Yao et al. 2025; Xu et al. 2024). There are also some studies on habitat selection by pika, but these mainly analyze altitude, habitat location, vegetation type, water sources, and soil properties (Ma 2011; Zhou, Hua, et al. 2013; Chang et al. 2021; Wang, Yan, Pawley, et al. 2024; Yang et al. 2024; Qi et al. 2024). Geomorphic and soil characteristics have been shown to influence the energy expenditure during burrow construction (Zelová et al. 2010), thermal comfort within burrows (Wei et al. 2013), and vegetation conditions (Tang et al. 2024). These factors collectively determine the quality of pika habitats (Du et al. 2019; Song 2022; Hua et al. 2020) and thus may directly shape pika habitat utilization preferences. However, there are still relatively few studies on how geomorphic and soil characteristics influence the preference for habitat utilization of pikas on a large scale, which is not conducive to objectively evaluating the environmental factors influencing the preference for habitat utilization of pikas, thereby limiting our understanding of how pikas adjust their adaptive behaviors to cope with the harsh plateau environment, and hindering the development of more effective management strategies.

In order to better understand the geomorphological and soil characteristics that influence the preference of pikas for habitat utilization, we performed field investigations of pika habitats with different population densities in three counties (Maqu, Luqu, and Xiahe) in the Gannan region to answer the following two questions: (1) Are the factors affecting pikas' habitat‐utilization preferences similar across regions? (2) If the first is true, is the habitat‐utilization preference of pikas related to geomorphic and soil characteristics, and what are the main factors influencing this preference? By addressing these questions, we hope to reveal the impact of geomorphic and soil characteristics on large scale habitat utilization preferences of pikas, thereby enhancing our understanding of how small mammals in high‐altitude areas respond to environmental changes.

## 2 Materials and Methods

### 2.1 Study Site

**FIGURE 2:**
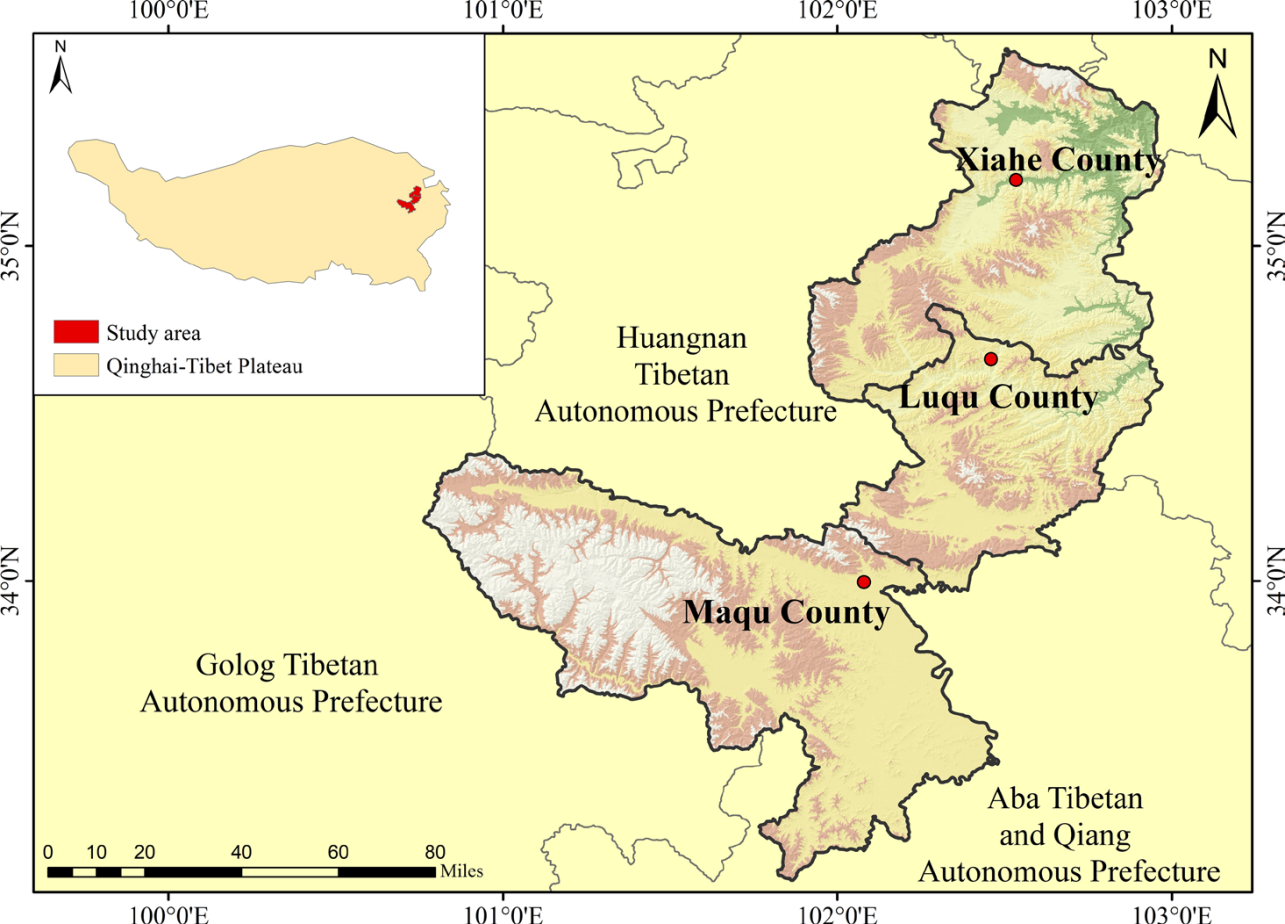
Locations of the study sites on the Qinghai‐Tibetan plateau. Map lines delineate study areas and do not necessarily depict accepted national boundaries.

The sample plots of this study are distributed across the counties of Xiahe, Luqu, and Maqu of the Gannan Tibetan Autonomous Prefecture (Figure 2). A total of 19 typical distribution areas for pikas were selected, such as Dajiutan in Xiahe County, Gahai and Shaiyintan in Luqu County, Azi Animal Husbandry Experimental Station, Hequ Racecourse, and Dashui Pasture in Maqu County. The local climate features large diurnal temperature differences, low temperatures, a short frost‐free period, and long sunshine duration, which is typical of an alpine continental habitat. The average altitude is between 3300 and 3600 m, and the average annual precipitation is between 446 and 586 mm. The grassland type is alpine and subalpine meadow. There are differences in the topography and soil conditions among each county. The vegetation in this area is dominated by Poaceae, Cyperaceae, Ranunculaceae, and Compositae, such as *Kobresia pygmaea*, *Elymus nutans griseb*, *Cremanthodium lineare*, *Anemone rivularis*, *Leontopium leontopodioides*, *Anemone rivularis, Trollius chinensis*, *Gentiana dahurica Fisch*, and *Leontopodium alpinum*.

### 2.2 Sample Plot Setting and Survey

From April to May 2011, we established a total of 38 survey points in Maqu County, Luqu County, and Xiahe County. Analysis revealed that the data volume was insufficient; therefore, an additional 28 survey points were added in April 2016. In total, we set up 66 pika monitoring points across the three counties, with 37 in Maqu County, 23 in Luqu County, and 6 in Xiahe County. The sample plots were established based on the typical distribution range of pikas and practical field accessibility (pikas are distributed in alpine meadows and alpine steppes at elevations above 3300 m). Maqu County is one of the primary distribution areas for pikas in the Gannan region, justifying the larger number of sample plots set up in this region. In contrast, Luqu County and Xiahe County have relatively fewer distribution points of pikas, resulting in the comparatively smaller number of sample plots. The size of the pika plot was 50 × 50 m and was selected on the basis of clear evidence of pika activity, such as fresh feces, such as we could directly observe the activity of pikas on the surface (Sun et al. 2015; Smith and Foggin 1999). The total burrow density of pikas ranged from a minimum of 656 burrows per hectare to a maximum of 2576 burrows per hectare in the surveyed plots of this study. The distance between each plot exceeded 5 km to ensure independence among the plots and to encompass the typical distribution range of the pikas. We investigated the total number of burrows in each plot to evaluate the population density of pika, as the total number of burrows is positively correlated with the absolute density of pika (Wei, He, et al. 2020). To investigate the soil properties, we randomly collected nine soil samples from the top 10 cm within a single plot. To reduce errors associated with soil heterogeneity caused by pika disturbance, all samples of the same plot were mixed thoroughly. The mixed soil samples were then evenly divided into three parts and stored separately in sealed bags with labels and brought back to the laboratory for soil moisture, soil texture and soil organic matter analysis.

The geomorphic characteristics of pika habitat included geomorphic types, slope features and water sources. We divided the landform into four categories: beach, terrace, valley and mountain (Wei 2013; Liu and Liu 2002; Zhang et al. 2016; Zhang et al. 2025). The criteria were as follows: “beach” refers to landform with flat terrain and no obvious line of sight obstacles within a radius of > 2 km, “terrace” refers to flat platform landform on both sides of a river, a “valley” is a relatively flat landform on either side of a valley stream, “mountain” refers to landform with slope > 10°. Slope features included slope aspect and slope degree, measured by compass and clinometer, respectively. The slope aspect was divided into east, south, west, north, southeast, southwest, northeast and northwest. There were four grades of slope: A, B, C and D, and the classification standard was Class A (0°–5°), Class B (6°–10°), Class C (11°–15°) and Class D (> 15°). We investigated the distribution of water sources around the habitat, including the type of water source and the distance from habitat to water. The type of water source was divided into two types according to whether the water source had flow: running or stagnant. The distance from habitat to water was calculated from the edge of the water source to the edge of the pika habitat. Soil depth refers to the distance between the surface and the parent material layer. Soil was drilled until the parent material layer was reached. The soil depth was measured at five random locations per site, and the mean of these samples was taken as the soil depth of the sample site. In this study, the soil texture was divided into four categories, sandy soil, loam, clay loam and sandy loam, according to the international standard of soil texture classification (Bormann 2010). Soil moisture content was measured using 100‐g soil samples by drying them for 24 h at 105°C in the laboratory. The soil organic matter content was determined using the Walkley‐Black wet combustion method (Nelson and Sommers 1996).

We used Vanderloeg and Scavia resource selection index to assess the habitat preferences of pika using nine discrete variables (including landform, slope direction, slope degree, type of water source, distance from habitat to water, soil depth, soil texture, soil moisture content, and soil organic matter content in the utilized sample plots according to the following formulae and calculations) (Zhou, Jiao, et al. 2013). Resource selection ratio (*ωi*): *ωi* = *oi*/ *πi*, *πi* = *ai*/*a*+, where *oi* is the selection proportion in *i* resource class; *ai* is the available area of resource class *i* and *a +* the total resources that can be used. Resource selection coefficient: *Wi* = *ωi*/Σ*ωi*. Resource selection index: *Ei* = (*Wi*–1/*n*)/(*Wi* + 1/*n*), where *i* is the number of grade *i* resources and *n* the number of grades of a given resource (*i* = 1, 2, 3 … *n*). Resource selection index scores were grouped into six categories (−1 ≤ *Ei* ≤ 1) as follows (Zhou, Jiao, et al. 2013):

- *Ei* = 1, especially preferred, indicating a complete preference for the habitat characteristic.
- 0.1 < *Ei* < 1.0, preferred, indicating that pikas showed a slight to strong preference for the habitat characteristic.
- *Ei* = 0, randomly selected, indicating that pikas did not show any preference or avoidance of this habitat characteristic.
−0.1 < *Ei* < 0.1, almost randomly selected, indicating a very weak preference or avoidance of the habitat characteristic.
−1 < *Ei* < −0.1, not preferred, indicating a slight to strong avoidance of the habitat characteristic.
- *Ei* = −1, not selected, indicating complete avoidance of the habitat characteristic.

### 2.3 Data Analysis

Firstly, to verify whether there are similarities in the habitat utilization preferences of pikas in different regions, the permutational analysis of variance (PERMANOVA) was used to compare the geomorphological and soil characteristics of pika habitat utilization preferences in three study areas. Secondly, we used the resource selection index scores (*Ei*) to evaluate pika habitat‐utilization preferences for landform, slope aspect, slope degree, type of water source, distance between habitat and water, soil depth, soil texture, soil moisture, and soil organic matter. Given the high correlation among the nine variables, we first performed Principal Component Analysis (PCA) using SPSS 27 to reduce dimensionality (Han et al. 2025). Values were assigned to non‐numerical variables before conducting the PCA. For the slope direction, the northern slope was assigned ‘−1’, the northwestern and northeastern slopes were assigned ‘−0.5’, no slope was assigned ‘0’, the southwestern and southeastern slopes were assigned ‘0.5’, and the southern slope was assigned ‘1’ (Yuan et al. 2024). This numerical assignment reflects the gradient of solar insolation and heat availability, with southern slopes receiving the most sunlight and northern slopes the least, which is ecologically relevant for microclimate and vegetation growth in alpine environments. We assigned ‘1’, ‘2’, ‘3’, and ‘4’ to Sandy soil, Sandy loam, Loam, and Clay loam according to the percentage of soil clay in the soil texture classification, respectively. It should be noted that landform (mountain, valley, terrace, and beach) and the type of water source (running water sources and stagnant water sources) were the categorical variables, and we used dummy‐variable encoding. We first centered and scaled the data, computed the sample correlation matrix, and obtained its eigenvalues and eigenvectors. Principal components and their contribution rates were then derived, and components with eigenvalues ≥ 1 were retained (Karlis et al. 2003). The main ecological factors relevant to the habitat utilization preferences of pikas were identified by PCA. High loadings (> 0.6, < −0.6) of variables onto the factors were used for subsequent factor interpretation. Lastly, this study constructed a Generalized Additive Model (GAM) to further investigate which environmental factors play a primary role in the habitat‐use preference of pikas (Li et al. 2017). Restricted Maximum Likelihood (REML) estimation was used to explain variations in burrow density (Link function = log, distribution family = negative binomial distribution), with burrow density serving as the response variable and habitat principal components as the explanatory variables. All GAM modeling was performed using the ‘gam ()’ function from the mgcv package (Wood 2017) in R (Rakotondravony et al. 2023).

## 3 Results

### 3.1 The Habitat Utilization Preferences of Pika Between Study Regions

The multivariate homogeneity of the three study areas was tested by the function betadisper before performing PERMANOVA, which indicated that there was no difference in dispersion between three study areas (*F* = 0.056, *p* = 0.946). The results of PERMANOVA also showed that there were no significant differences in the geomorphic and soil characteristics of pika habitat utilization preferences among the three study regions (*F* = 0.936, *p* = 0.477, *R* ^2^ = 0.02887). This indicates that the topography and soil characteristics of the habitat utilization preferences of pikas may potentially be similar. Therefore, we integrated data from the three study regions to analyze the preferences of pika habitat utilization for geomorphic and soil characteristics.

### 3.2 The Utilization Preference of Pika Habitat Is Related to Geomorphic and Soil Characteristics

The pika has a clear preference for geomorphic and soil characteristics in habitat utilization (Table 1). Of the four types of landforms, pikas prefer to use beach (*Ei* = 0.205), randomly select terraces (*Ei* = 0.043), and valley (*Ei* = −0.015), and avoid mountain (*Ei* = −0.404). In the seven observed mountain landforms, the slope aspect of the pika habitat was directed towards the sunny and semi‐sunny slopes (south, southeast, and southwest), but with a clear preference for south‐facing slopes (*Ei* = 0.26). The slope of all the observed plots was below 16°, but pikas prefer habitats with slopes < 5° (*Ei* = 0.525), while avoiding habitats with slopes > 5° (11°–15°: *Ei* = −0.886; 6°–10°: *Ei* = −0.158). The type of water source also affects the habitat utilization preferences of pikas; they exhibit a strong preference for running water sources (*Ei* = 0.175) and actively avoid stagnant water sources (*Ei* = −0.269). After categorizing the types of water sources, we found that pikas prefer habitats located < 200 m away from running water sources (101–200 m: *Ei* = 0.342; 0–100 m: *Ei* = 0.457), and habitats located between 200 and 400 m from the stagnant water sources (201–300 m: *Ei* = 0.479; 301–400 m: *Ei* = 0.224). Soil texture affects the habitat utilization preferences of pikas; they prefer loam (*Ei* = 0.258) and avoid sandy soil (*Ei* = −0.692). Pikas prefer soil moisture content between 10% and 20% (*Ei* = 0.29), soil organic matter content between 5% and 15% (*Ei* = 0.359), and soil depth between 40 and 60 cm (40–50 cm: *Ei* = 0.214; 50–60: *Ei* = 0.241), while avoiding habitats with soil moisture content < 10% (*Ei* = −0.2) and more than 20% (*Ei* = −0.35), soil organic matter content < 5% (*Ei* = −0.633) and more than 20% (*Ei* = −0.739), and soil depth between 20 and 30 cm (*Ei* = −0.695) and more than 70 cm (*Ei* = −0.375).

**TABLE 1:** Habitat utilization preferences of the plateau pika.

Habitat variables	*I*	*Wi*	*Ei*	Selection
Landform (LF)	Beach (B)	0.379	0.205	P
	Terrace (T)	0.273	0.043	AR
	Valley (V)	0.242	−0.015	AR
	Mountain (M)	0.106	−0.404	NP
Slope aspect (SA)	South	0.061	0.260	P
	Southeast	0.030	−0.080	AR
	Southwest	0.015	−0.400	NP
Slope degree (SD)	0°–5°	0.803	0.525	P
	6°–10°	0.182	−0.158	NP
	11°–15°	0.015	−0.886	NP
	> 15°	0	−1	NS
Type of water source (TWS)	Running water sources (RWS)	0.712	0.175	P
	Stagnant water sources (SWS)	0.288	−0.269	NP
Distance from habitat to running water sources (RWS‐DHW)	< 100	0.340	0.343	P
	100–200	0.447	0.457	P
	201–300	0.085	−0.324	NP
	301–400	0.043	−0.593	NP
	401–500	0.043	−0.593	NP
	> 500	0.043	−0.593	NP
Distance from habitat to stagnant water sources (SWS‐DHW)	< 100	0	−1	NS
	100–200	0.105	−0.226	NP
	201–300	0.474	0.479	P
	301–400	0.263	0.224	P
	401–500	0.158	−0.027	AR
	> 500	0	−1	NS
Soil texture (ST)	Loam (L)	0.424	0.258	P
	Clay loam (CL)	0.258	0.015	AR
	Sandy loam (SL)	0.273	0.043	AR
	Sandy soil (SS)	0.045	−0.692	NP
Soil moisture (SM)	0–10	0.167	−0.200	NP
	10.1–20	0.455	0.290	P
	20.1–30	0.258	0.015	AR
	> 30	0.121	−0.350	NP
Soil organic matter (SOM)	0–5	0.045	−0.633	NP
	5.1–10	0.424	0.359	P
	10.1–15	0.333	0.250	P
	15.1–20	0.167	−0.090	AR
	> 20	0.030	−0.739	NP
Soil depth (SDT)	20–30	0.030	−0.695	NP
	31–40	0.182	0.043	AR
	41–50	0.258	0.214	P
	51–60	0.273	0.241	P
	61–70	0.182	0.043	AR
	> 70	0.076	−0.374	NP

Abbreviations: AR, Almost random selection; *Ei*, Selection index; EP, Especially preferred; *I*, Index of Characteristics; NP, Not preferred; NS, Not selected; P, Preferred; RS, Random selection; *Wi*, Selection coefficient.

### 3.3 Principal Geomorphological and Soil Factors Affecting the Preferences of Pika Habitat Utilization

**FIGURE 3:**
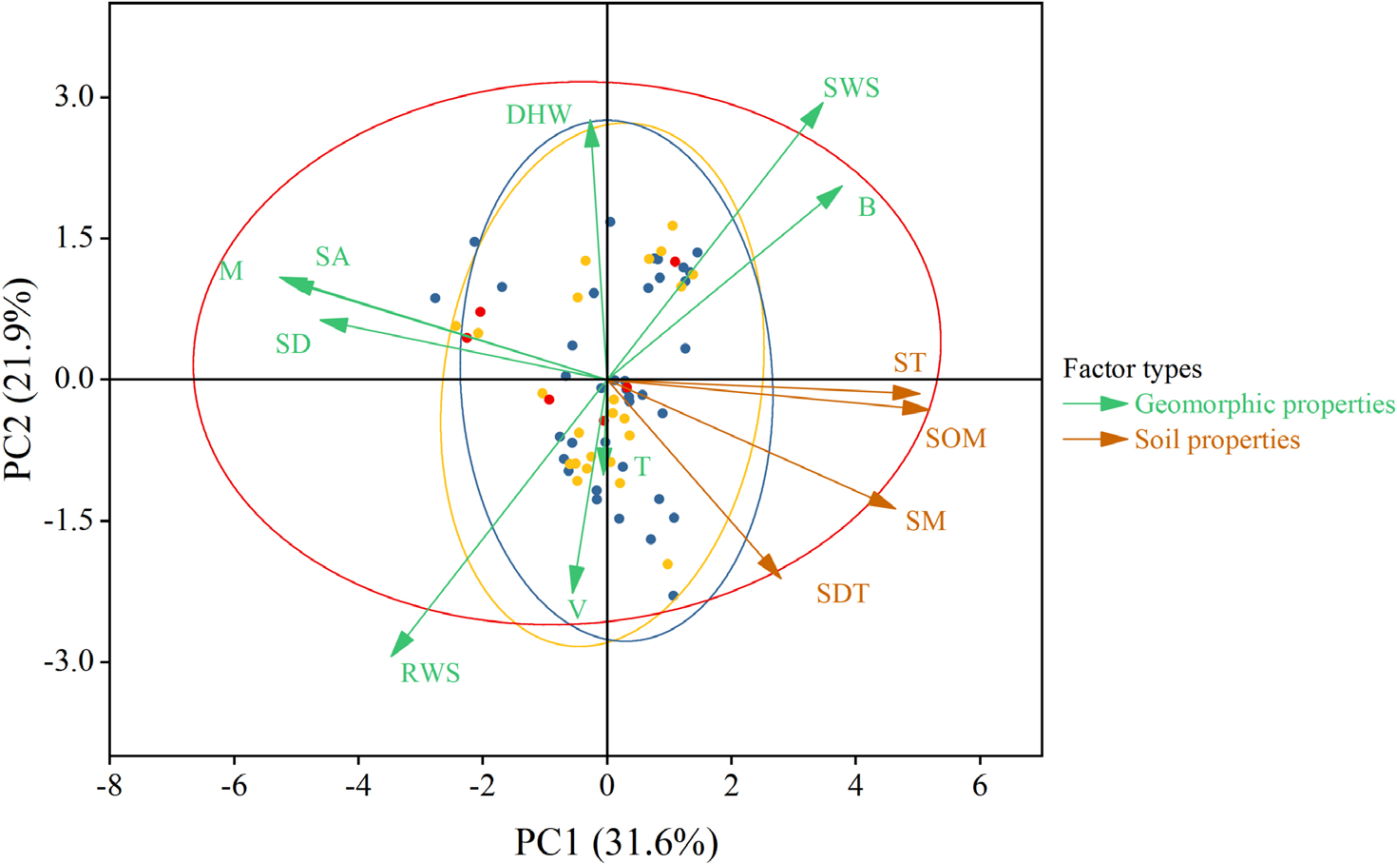
Principal component analysis (PCA) of the plateau pika habitat utilized landform and soil variables in three counties. The red color represents Xiahe county, blue represents Maqu county, and yellow represents Luqu county. Abbreviations of variables are the same as those in Table 1.

The PCA was conducted to develop an integrated geomorphic and soil habitat utilization index for the pika. In the PCA, the first four principal components covered 78.487% of the variance with a *p*‐value of < 0.01. The first principal component (PC1) explained 31.646% of the variance. It positively correlated with soil organic matter (0.752), soil texture (0.729), and soil moisture (0.672), but negatively correlated with mountain (−0.763), slope aspect (−0.748), and slope degree (−0.668) (Table 2). The second principal component (PC2) explained 21.911% of the variance. It was positively correlated with stagnant water sources (0.744) and the distance between habitat and water (0.698) but negatively correlated with running water sources (−0.744) (Table 2). The third principal component (PC3) explained 14.119% of the variance. It was positively correlated with soil texture (0.597) (Table 2). The fourth principal component (PC4) explained 10.811% of the variance. It was negatively correlated with terrace (−0.955). Among these, the loadings of soil depth on the four principal components were all below 0.6 or above −0.6 (specifically 0.405, −0.535, −0.01, and 0.044) (Table 2). The biplot of the habitat parameter PCA showed that stagnant water sources and beach were positively related to both PC1 and PC2, while running water sources, valley, and terrace were negatively related to both PC1 and PC2 (Figure 3). Running water sources, slope degree, slope aspect, and mountain were only positively related to PC2, while soil organic matter, soil texture, soil moisture, and soil depth were positively related to PC1 (Figure 3). Overall, geomorphic and soil characteristics are factors that affect the habitat selection of pikas.

**TABLE 2:** The load matrix of the principal component.

Factor	PC1	PC2	PC3	PC4
B	0.548	0.521	0.092	0.358
M	**−0.763**	0.275	0.490	0.103
T	−0.009	−0.256	−0.118	**−0.955**
V	−0.08	−0.574	−0.361	0.545
SA	**−0.748**	0.267	0.499	0.104
SD	**−0.668**	0.161	0.472	−0.131
RWS	−0.503	**−0.744**	0.175	0.080
SWS	0.503	**0.744**	−0.175	−0.080
DHW	−0.039	**0.698**	−0.208	−0.006
ST	**0.729**	−0.038	**0.597**	0.037
SM	**0.672**	−0.347	0.470	0.038
SOM	**0.752**	−0.081	0.537	−0.109
SDT	0.405	−0.535	−0.01	0.044
Eigenvalue	4.114	2.848	1.835	1.405
Proportion of variance	31.646	21.911	14.119	10.811
Cumulative proportion	31.646	53.557	67.676	78.487

*Note:* Abbreviations of variables are the same as those in Table 1. Bold values indicate high factor loadings (> 0.6 or < −0.6).

**FIGURE 4:**
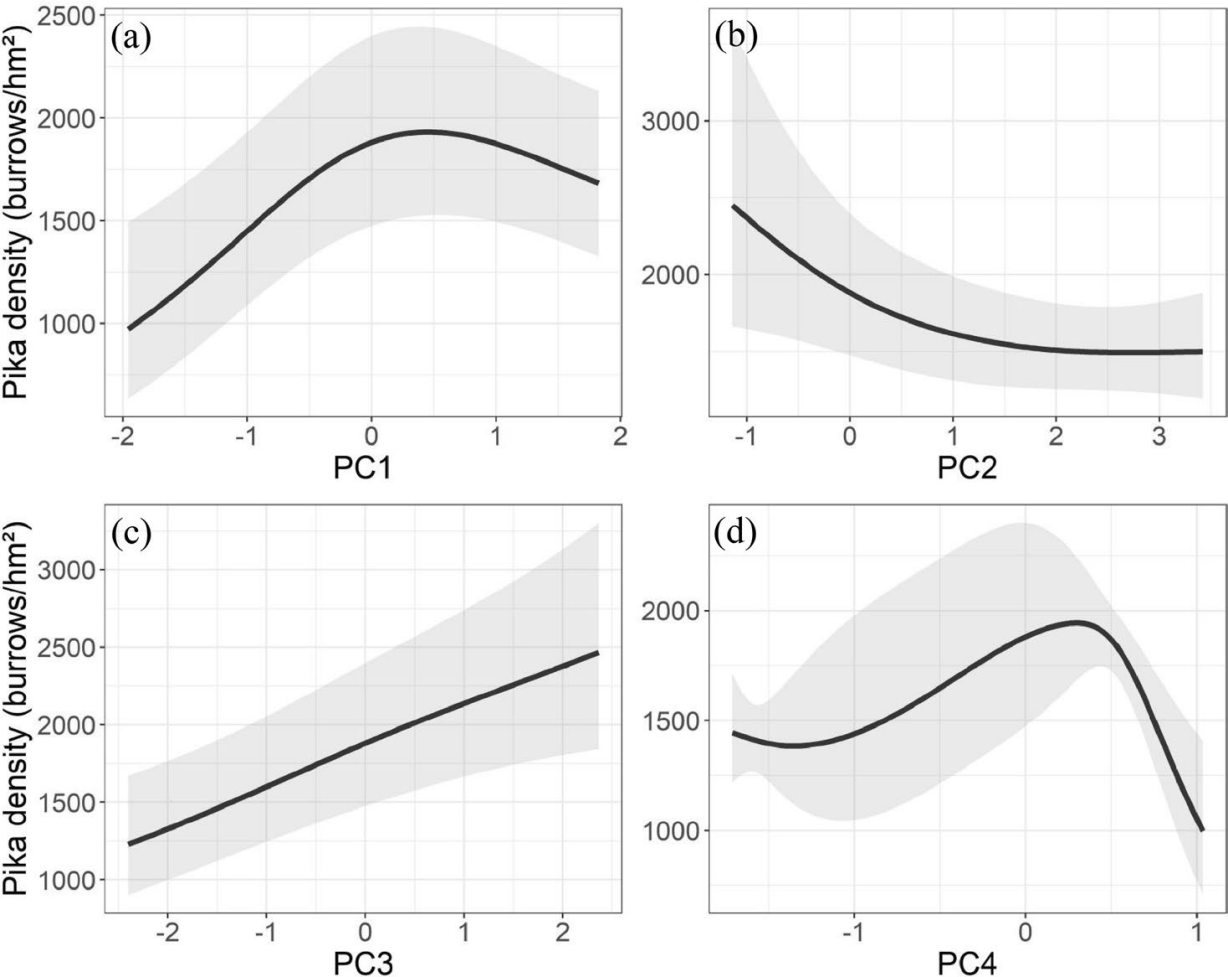
The relationship between four principal components and pika density. The solid lines represent the fitted curves for each independent variable, and the gray areas indicate the 95% confidence intervals.

The multivariate generalized additive model (GAM) analysis based on four principal components revealed that all four principal components influenced pika population density. Among them, the effects of PC3 and PC4 were extremely significant, while the effects of PC1 and PC2 were significant (Table 3). Figure 4 illustrates the fitted curve of each principal component in the GAM model. It can be observed that the greater the degree of freedom, the greater the fluctuation in the fitted curve. Among all the fitted curves, there is a correlation between all four principal components and pika population density. These relationships varied among the independent variables: On PC1, which is dominated by soil factors (soil texture, soil moisture, soil organic matter) and topographic factors (slope aspect, slope degree), and on PC1 and PC4, which are dominated by geomorphic factors, pika population density showed a trend of first increasing and then decreasing. This unimodal response indicates that pikas prefer to use habitats with flat terrain and moderate soil moisture and organic matter. The PC2 axis mainly reflects changes in water source factors. As PC2 shifts from running water sources to stagnant water sources, and as the distance to water sources increases, pika population density shows a significant negative correlation with the water source factor (PC2) within a certain range. This indicates that as habitats become farther from flowing water sources, pika population density declines. Soil texture (PC3) has a significant positive correlation with pika population density, indicating that pikas prefer to use soil types with high clay content.

**TABLE 3:** Results of the generalized additive model (GAM) using four principal components to explain variations in pika burrow density.

Term	Estimate	Std. Error	edf	Ref.df	*χ* ^2^	*p*	Sig.
(Intercept)	7.266	0.019	—	—	—	< 0.001	***
s(PC1)	—	—	1.927	1.988	14.589	0.001	**
s(PC2)	—	—	1.701	1.896	7.839	0.013	*
s(PC3)	—	—	1.550	1.789	29.417	< 0.001	***
s(PC4)	—	—	3.243	3.708	20.173	< 0.001	***

Abbreviations: edf, estimated degrees of freedom; Ref.df, reference degrees of freedom; s(), smooth function term; Std. Error, standard error; *χ* ^2^, Chi‐square test statistic. Significance codes: ***p < 0.001, **p < 0.01, *p < 0.05.

## 4 Discussion

Animals' habitat utilization preferences represent a key adaptive strategy for coping with environmental changes and are influenced by multiple factors. Suitable habitat is essential to ensure survival and reproduction of animals, especially in high plateau areas with harsh environmental conditions (Danielson 1992; Avila‐Flores et al. 2010; Olimb et al. 2022; Little et al. 2022). To adapt to the severe and variable environmental conditions of the QTP, pikas select habitats based on specific geomorphic and soil factors. The results of this study showed that there was a convergence of factors affecting the habitat utilization preferences of pikas in different regions. In addition, our results from *Ei*, PCA and GAM all indicated that geomorphic and soil properties affect the habitat utilization preferences of pika, except soil depth.

The results of the PERMANOVA suggest that the geomorphic and soil characteristics driving pika habitat utilization are potentially consistent across the studied regions. However, this does not indicate that pikas choose these environmental factors, and more due to the similarity of the characteristics of environmental variables (geomorphic and soil) in the habitats of pikas in different regions of the QTP (Hu et al. 2020), because the harsh natural environment of the QTP imposes strong niche constraints on the survival of pikas (Zhou et al. 2024). Therefore, we speculate that the constraints imposed by natural environmental conditions lead to pikas living in similar environments in different regions.

The results of the resource selection index (*Ei*) showed that pikas have a clear preference for geomorphic and soil characteristics in habitat utilization. Similarly, the results of PCA showed that the cumulative contribution of the first four principal components was 78.487%, which can reflect the habitat characteristics of habitat use preferences of pikas. The first four principal components all influenced the habitat use preferences of pikas. In addition, the results of the GAM were consistent. The plateau topography is complex, dominated by undulating mountains, and has rare plains in QTP (Hu et al. 2020). Our results suggest that pikas prefer beach, and this could be explained by the following: first, the soil layer in the mountain is very thin, which is not conducive to the survival of pikas in winter (Wei et al. 2013). Second, landform and soil directly determine the quality of their habitat, living environment, and other basic factors, and affect vegetation types; thus, mountain landforms indirectly affect their food abundance and quality (Jiang 1998). Numerous studies have shown that pikas prefer flat terrain with a wide field of view (Wei, Zhen, et al. 2020; Wei et al. 2022), which is mainly related to their strategy to avoid predation risk (to facilitate timely detection of predators) (Zhang, Zhang, et al. 2005). Among the geomorphic types in this study, the only categories that can relatively meet the requirements of “flat terrain and broad field of vision” were beach. In addition, pikas prefer habitats with slopes < 5°, and the relationship between PC1, PC4, and pika density both indicate that pikas do not prefer mountains and terraces with excessive slopes (Figure 4a,d), which also supports the above results. Slope direction is a key factor in the redistribution of water and heat in a particular niche and directly determines the type of vegetation, which determines whether small herbivorous mammals have sufficient food resources. In this study, pikas preferred south‐facing slopes. This selection orientation may be related to food resources indirectly represented by vegetation types. Firstly, south‐facing slopes receive more direct solar radiation, leading to earlier snowmelt in winter, which makes it easier for pikas to access food (Liu et al. 2010). Secondly, plants on sunlit slopes green up earlier in spring compared to those on shaded slopes (Li et al. 2021), providing ample food resources for pikas during their breeding season. Additionally, winter in high‐altitude areas can last up to 7 months, and the thin soil layer on mountain landforms can lead to lower temperatures in the burrows of pikas. Therefore, the choice of the southern slope in mountain landforms may reflect the special needs of high‐altitude lagomorphs for sunlight, temperature, and food resources (Wei et al. 2013).

Water source is an important determining factor in a wild animal's habitat utilization preferences (Ma 2011). The availability of water and distance to water are usually the primary considerations for habitat utilization preferences in wild animals (Smith 1986). In this study, our results indicated that pikas prefer to use habitats with running water sources, and this was supported by the relationship between PC2 and pika density (Figure 4b). The rainfall on the QTP is mainly concentrated from June to September, and short‐term heavy rainfall can easily form runoff. Stagnant water sources in low‐lying areas cannot discharge runoff in a timely manner, which can pose a threat to the survival of pikas in the surrounding areas. Pikas prefer habitats located closer to running water sources, but further from stagnant water sources, indicating that pikas distinguish between the types of water sources and alter the distance of their habitat according to these water sources (Table 1). Currently, no drinking behavior has been reported for pikas in their natural habitats. Furthermore, studies have found that pikas are distributed even in areas far from water sources (beyond their typical activity range) (Song 2022), indirectly supporting the possibility that pikas may lack a habit of drinking water. Observations by visiting herdsmen and the personal experience of the authors throughout the year also do not indicate that pikas drink free water, possibly because the water content in their food is sufficient to maintain their normal life activities. Unlike large herbivores, pikas occupy relatively fixed habitats and do not migrate (Redfern et al. 2005). Therefore, their preference for the distance to different types of water sources reflects a comprehensive assessment of local conditions. First, running water sources readily generate runoff during the rainy season; however, the habitats of pikas near such sources are less affected by surface runoff. In contrast, the avoidance of habitats close to stagnant water sources underscores this preference. Second, regions close to water sources generally have higher soil moisture content and more lush vegetation, which provides pikas with richer and higher‐quality food resources (Xu and Xue 2013). Third, areas near water sources typically have deeper soil layers, meeting the soil conditions required for pikas to construct burrows. Therefore, the observed preferences for distance between habitat and water resources and running water sources in pikas reflect a resource‐risk trade‐off in their habitat utilization strategy.

The soil is the primary location of nests for burrowing animals and plays an important role in determining where they will occur (Ma 2011; Wei 2013). Soil texture directly affects the stability and robustness of the nest structure (Wei et al. 2013). Of the four soil types, loam has the best soil conditions in terms of firmness and stability (Xin et al. 2018). Sandy soil and sandy loam are not very stable or firm, and the loose configuration of the soil also precludes heat preservation. Clay loam preserves heat very well but is too compact and hard for easy digging and increases the energy consumption of the pikas. Therefore, only loam meets the basic requirements of pikas in terms of tunnel stability, firmness and heat retention (Wei et al. 2013). Our results confirm that pikas preferentially burrow in loam. However, it is not necessarily the case that the more clay particles there are, the better. The more clay particles there are, the higher the energy consumption of pikas digging burrows, especially those complex permanent burrows. The relationship between PC1 and pika density (Figure 4a) further illustrates this optimized selection, indicating that pikas prefer an “optimal” soil texture. This reflects a strategic trade‐off between energy expenditure for burrow construction and burrow stability. The thickness of the soil layer is a prerequisite for determining whether the geomorphic environment is suitable for the construction of the tunnel system. If the soil layer is too thin, the tunnel system will not be warm, stable, or safe enough (Wei 2013). It therefore will not be suitable for inhabitation, even if other conditions such as food availability are good. The result of *Ei* showed that pikas preferred habitats with soil depth between 40 and 60 cm. However, the relationship between PC (1, 2, 3, and 4) and pika density indicated that there was no correlation between soil depth and pika density. Of course, construction of nests by pikas requires consideration of soil depth, which determines heat retention (Clémentine et al. 2010), especially in winter (Wywialowski 1987). Potential explanations for this seemingly contradictory result are firstly, pikas can only construct permanent burrows when the soil layer reaches a certain depth, but deeper soil layers would lead to excessive energy expenditure (Zhang and Liu 2024; Li et al. 2025). Moreover, the relatively short soil development time on the QTP generally results in thinner soil layers (Wang et al. 2002), which may lead to an insufficient actual range of variation in soil depth to demonstrate statistically significant associations in the results. Finally, habitat utilization preferences are the result of the combined effects of multiple factors. Once the basic requirements for soil depth are met, other factors (such as soil moisture and soil organic matter) may become more prominent drivers of pika density. Hence, there is no causal relationship between the soil layer depth and the presence of pika habitat (Wang et al. 2004).

From the perspective of small‐scale topographic and geomorphic selection, a preference for high and dry terrain is accepted for pikas (Fan and Zhang 1996; Wei et al. 2013). This study verified this conclusion from the perspective of the soil moisture content. Pikas prefer habitats with soil moisture content between 10% and 20%, while avoiding habitats with soil moisture content < 10% and more than 20%. In addition, this was supported by the relationship between PC1 and pika density (Figure 4). This may be to maintain the temperature and humidity inside the nests within a certain range. Low soil water content leads to dry soil, and the temperature in the nest can be susceptible to influence from the external plateau climate (Fan and Zhang 1996). High soil moisture leads to higher humidity in the nest, and the high humidity environment can be conducive to microbial growth (Fan and Zhang 1996). Therefore, too low or too high soil moisture will affect the survival of pikas. The level of soil organic matter is an important factor for assessing soil fertility, and soil organic matter is an indicator of the habitat suitability and selection orientation of pika. The results of this study showed that pikas have a clear preference for habitats with 5%–15% soil organic matter and avoid soil environments with soil organic matter more than 20%. The relationship between PC1 and pika density yielded similar results (Figure 4a). This preference reveals the complex trade‐off between foraging needs and anti‐predator risks in pikas. Soil characteristics with high soil organic matter are conducive to the growth of tall grasses. High grass cover increases the predation risk of pikas in this habitat (Dobson et al. 2000), which is not conducive to fitness for pikas. This suggests that decreasing predation risk is a key driver of pika habitat selection. The avoidance of soil environments with soil organic matter content < 5% may be related to dietary requirements of the pika. Low soil organic matter soils are typically less fertile and unfavorable for the growth of graminoids, which constitute the primary food source of pikas (Niu et al. 2019; Dong et al. 2025). Therefore, selecting habitats with moderate soil organic matter content may reflect a trade‐off between food availability and predation risk.

Our findings further demonstrate that the habitat utilization preferences of non‐hibernating rodents in high‐altitude areas result from an optimal trade‐off among various influencing factors. This indicates that animal habitat preferences are not merely passive responses to available resources (Dai et al. 2007) but rather represent active adaptive strategies to environmental conditions (Asres and Amha 2014). Moreover, with the intensification of global climate change (Yang et al. 2022; You et al. 2021; Song et al. 2025), rising temperatures and increased precipitation on the QTP may facilitate the expansion of pika ranges to higher elevations. The results of this study can provide a basis for identifying areas prone to pika infestation under future climate change.

Although this study preliminarily reveals the habitat utilization preferences of pikas regarding geomorphic and soil characteristics at a regional scale, some limitations remain. First, the study area only covers three counties in the Gannan region along the eastern edge of the QTP (elevations ranging from 3345 to 3800 m), which may not fully represent the habitat utilization preferences of pikas across the entire plateau. Second, we did not measure soil moisture at depths of 10–20 cm (where pika nests are typically located), which limits our understanding of the relationship between soil moisture and pika burrow depth.

## 5 Conclusion

The results of this study indicate that the geomorphic and soil characteristics of the habitat‐utilization preferences of pikas are potentially similar across different regions. In addition, the results clearly suggest that pikas have a strong preference for geomorphic and soil characteristics and that landform, slope aspect, slope degree, type of water source, distance between habitat to water, soil depth, soil texture, soil moisture, and soil organic matter were all factors affecting pika habitat‐utilization preferences. Consistent with previous studies, pikas preferred flat terrain and a wide field of view (beach) and had a selective slope direction for mountainous types. Our results indicated that pikas preferred habitat with running water sources. In addition, pikas had a preference for loam, soil depth > 40 cm but < 60 cm, and for soil organic matter and soil moisture values between 5%–15% and 10%–20%, respectively. These habitat‐use preferences are not merely passive responses but rather represent behavioral adaptation strategies employed by pikas in response to environmental changes. Given ongoing environmental change, understanding the habitat use preferences of pikas is crucial for formulating effective management policies.

## Author Contributions

**Faming Qin:** formal analysis (equal), investigation (equal), visualization (equal), writing – original draft (equal), writing – review and editing (equal). **Yingyuan Chen:** investigation (equal), writing – review and editing (equal). **Zhongmin Tang:** investigation (equal), writing – review and editing (equal). **Maria K. Oosthuizen:** writing – review and editing (equal). **Shenghui An:** investigation (equal), writing – review and editing (equal). **Wanrong Wei:** conceptualization (lead), data curation (lead), formal analysis (equal), investigation (equal), visualization (equal), writing – original draft (equal), writing – review and editing (equal).

## Funding

This work was supported by the China Postdoctoral Science Foundation, 2023M731471; Natural Science Foundation of Sichuan Province, 23NSFSC5406; Natural Science Foundation of Gansu Province, 23JRRA1094, 26JRRA170.

## Conflicts of Interest

The authors declare no conflicts of interest.

## Supporting information


**Table S1:** Raw data of geomorphic properties, soil characteristics, and plateau pika burrow density (number of burrows/hm^2^) measured across the surveyed study sites.

## Data Availability

All the required data are uploaded as Supporting Information (Table S1).
